# Assessing the Preliminary Efficacy of a Nonrandomized, Clinic-Based HIV Risk Reduction Pilot Intervention for PrEP-Initiated, Alcohol- and Other Drug-Using Women of Color in Miami, FL

**DOI:** 10.1007/s40615-022-01482-5

**Published:** 2023-01-17

**Authors:** Amanda Ichite, Michèle Jean-Gilles, Rhonda Rosenberg, John Abbamonte, Jessy G. Dévieux

**Affiliations:** 1 Department of Health Promotion and Disease Prevention, Robert Stempel College of Public, Health and Social Work, Florida International University, Miami, FL 33199 USA; 2 Department of Educational and Psychological Studies, Counseling Psychology Area, University of Miami, Coral Gables, FL 33146 USA

**Keywords:** Pre-exposure prophylaxis, PrEP, PrEP barriers, PrEP facilitators, HIV prevention, Risk reduction intervention, Women of color, Mixed methods

## Abstract

In this paper, we assessed the preliminary efficacy and acceptability of a quasi-experimental, clinic-based sexual risk reduction pilot intervention for pre-exposure prophylaxis (PrEP)-initiated, alcohol- and other drug-using women of color and explored their self-reported barriers to, and facilitators of, PrEP use. Using a mixed methods design, analyses incorporated pre- and post-intervention study assessment data from 38 women and semi-structured process evaluations using a subsample of 25. The intervention administered over an 8-week period consisted of 4 one-on-one in person educational sessions, a process evaluation, and study assessments conducted at baseline and 3 and 6 months. Post intervention, statistically significant changes in sexual risk scores were not observed; however, we found significant decreases in alcohol use (*Z* =  − 3.02, *p* = .003, η^2^ = .41). Process evaluation data revealed interpersonal relationships as a key motivator for PrEP initiation as well as a prominent barrier to PrEP use; these relationships rarely facilitated adherence. Overall, women found the intervention to be acceptable and reported a wide range of benefits of participation—most notably its therapeutic benefits. Findings from this study provide preliminary evidence of the potential for the Talking PrEP with Women of Color intervention to improve risky behaviors, knowledge, and attitudes related to sexual risk taking. Furthermore, findings suggest that interventions to increase PrEP uptake and adherence in at-risk women may benefit from supporting them in accurately estimating their risk for HIV and increasing their sense of social support.

## Introduction

The HIV epidemic in the USA is characterized by disparities across dimensions of race/ethnicity, gender identity, sexual orientation, substance use patterns, and geographical region [1]. For decades, Black/African American people have accounted for the highest percentage of new HIV diagnoses in the USA—accounting for 42% of new diagnoses in 2020 [2]. This statistics holds true for Black women who made up 54% of new diagnoses and had the highest rate of HIV diagnoses (16.4/100,000 persons) among women in 2020—11 times the rate of new diagnoses in White women and 4 times the rate in Hispanic/Latina women [2]. These statistics suggest that effective HIV prevention methods are not reaching populations who could benefit from them most.

Understanding the factors that increase the vulnerability of Black women to HIV is imperative [3]. Individual level factors include the perceptions, attitudes, and beliefs of women (i.e., perceived susceptibility) [4, 5]. Studies have found that African American women continued to engage in unprotected sex with their male partners who they were aware had multiple sex partners [6]. This behavior has been associated with a lack of power in the relationship or an intentional gesture made to empower their partner [6]. Alcohol use is another prominent factor, as it interferes with the processing of information and decreasing perceptions of risk—leading to increased risk-taking [3, 7]. Drug use has been linked to behaviors such as transactional sex, concurrent sex partners, inconsistent condom use, and engaging in sex within high-risk social networks, all of which increase HIV risk [8]. Substance use is also associated with discontinuation of, or non-adherence to, effective HIV prevention medications such as pre-exposure prophylaxis (PrEP) [9, 10].

PrEP is an essential tool for reducing HIV risk in Black women—particularly risk associated with ineffective condom negotiation. It is the first 100% female-controlled HIV prevention tool that can be used discretely by women without the knowledge or involvement of their partners [11] and has the ability to reduce the risk of contracting HIV from vaginal intercourse by over 71% [12]. Despite the proven effectiveness, widespread availability, and apparent need for PrEP in women, rates of utilization are much lower than that in men—with 92% of all PrEP users in 2021 being men [13].

Previous studies have identified cost [11, 14–16], medical mistrust [11, 17], safety/side effects concerns [11, 14–16], HIV-related stigma [11, 15, 18], lack of family or partner support [11, 15, 17], low perceived efficacy in daily adherence [11, 17], poor patient-provider communication [15], and underestimation of HIV risk [14, 15, 17] to be common barriers to PrEP utilization and adherence in women. Focus groups exploring PrEP knowledge and attitudes among cisgender women found that the desire to maintain health, social support, reminders, and hearing about HIV from women living with HIV were facilitators to PrEP initiation and adherence [15]. A similar study found lack of social support to be a significant barrier to PrEP use [17]. Furthermore, previous studies found female sex to be linked with PrEP discontinuation [9, 19]. Retention in care is a fundamental factor in sustaining the population level impact of daily oral PrEP use [20, 21]. Given the challenges to engage and retain women on PrEP, research studies and implementation projects have incorporated strategies to support adherence using behavioral interventions [22]. These strategies are needed to continue increasing PrEP uptake and promoting persistence among high-risk populations (e.g., Black women in the South) [18].

### Background

There is considerable evidence supporting the efficacy of behavioral interventions in reducing HIV sexual risk behaviors; however, the growing consensus is that simply providing information as a behavioral health intervention is not an effective means of achieving sexual behavior change [23]. A review of recent HIV prevention interventions revealed that future interventions to increase PrEP uptake may be most effective if they provide a combination of PrEP-related information, personal and social motivation, and practical behavioral skills associated with PrEP use (i.e., adherence, negotiation, and managing side effects) [24].

The development of the “Talking Prep with Women of Color (WOC) in Miami” intervention was guided by the Information-Motivation-Behavioral Skills (IMB) model of PrEP uptake. This model is an adaptation of the traditional IMB [25]. The IMB model of PrEP uptake asserts that individuals at risk for HIV will overcome obstacles to initiate and adhere to PrEP if they have sufficient PrEP information, are motivated to act on that information, and are equipped with the behavioral skills essential for seeking out and initiating PrEP [24]. Although a number of interventions have aimed to reduce risky sexual behaviors in women, “Talking PrEP with Women of Color in Miami” is the first intervention of its kind in South Florida using the IMB model of PrEP uptake to reduce risky behaviors in at-risk ethnic minority women.

### Present Study

Using longitudinal study assessment data and qualitative, post-intervention process evaluation data, this paper assesses the preliminary efficacy and acceptability of the pilot PrEP intervention “Talking PrEP with Women of Color in Miami” on HIV risk behaviors in a sample of PrEP-initiated, alcohol- and other drug-using WOC at high risk for HIV. This paper also assesses the self-reported barriers and facilitators of PrEP use.

## Methods

### Study Design and Setting

Secondary data analysis was conducted on this quasi-experimental study using a pre- and post-longitudinal design, to conduct an exploratory pilot among WOC receiving PrEP care at a publicly funded community health center (CHC) in Miami, FL. The study aimed to evaluate the feasibility, acceptability, and fidelity of a multi-component evidence-based intervention to reduce health disparities in engagement, utilization, and retention in PrEP care among African American, Latina, and Haitian women.

The study site, located in an area with the highest number of people living with HIV/AIDS in Miami Dade County (Zone IV) [26], offered a variety of primary medical, HIV prevention, and HIV specialty care services to a largely underserved population burdened by high rates of poverty, homelessness, lack of insurance, and unaddressed mental health and substance abuse issues.

### Ethics Statement

This study was approved by Florida International University’s Institutional Review Board. The parent study, “Optimizing PrEP Utilization Among Alcohol and Other Drug Using Women of Color,” from which this study’s data was sourced, was funded by award Number U34AA026219 from the National Institute on Alcohol Abuse and Alcoholism. All women provided verbal and written informed consent prior to participation.

### Inclusion/Exclusion Criteria

Participants were multiethnic women between the ages of 18 and 45 who were enrolled in the CHC’s PrEP Program. To be included in the intervention study, women had to be cisgender African American, Latina, or Haitian women with no major unaddressed mental health issues and not living with HIV. They also had to have initiated PrEP at the CHC no more than 1 month prior to their study enrollment date and have taken at least 1 dose of PrEP before officially being enrolled.

### Recruitment and Enrollment

Study participants were recruited from the CHC’s PrEP Program from May 2019 to May 2021. Once a woman was prescribed PrEP, she was asked if she was interested in participating in a research study concerning PrEP. Interested patients consented to having their contact information released to the study interventionist. The interventionist followed up with potential participants in person or via telephone to provide further details about the study, screen for study eligibility, and schedule the initial study visit. If time permitted and all enrollment criteria were met, patients who were enrolled in person completed the baseline assessment. As recommended by the Transparent Reporting of Evaluations with Nonrandomized Designs statement guidelines, [27] a flow diagram was created to show the number of the participants through each stage of the study.

### Intervention

The pilot PrEP intervention, “Talking PrEP with Women of Color in Miami,” was created by a Community Advocacy and Advisory Board consisting of researchers, clinicians, community organizers, and lay community members as part of a multitiered intervention program for South Florida. The aim was to address HIV in women and girls through community-based participatory research. Developed using basic elements of motivational interviewing (MI) and a strengths-based approach, this manualized intervention sought to reduce sexual risk behaviors and support adherence among PrEP-initiated minority women. The intervention was intended to be delivered one-on-one in a community-based setting by a trained peer-level lay worker, called the PrEP Master. The PrEP Master was equipped with a manual with scripted educational and phone sessions, and was encouraged to adapt language as needed to match the level of the participant, when necessary.

**Fig. 1:**
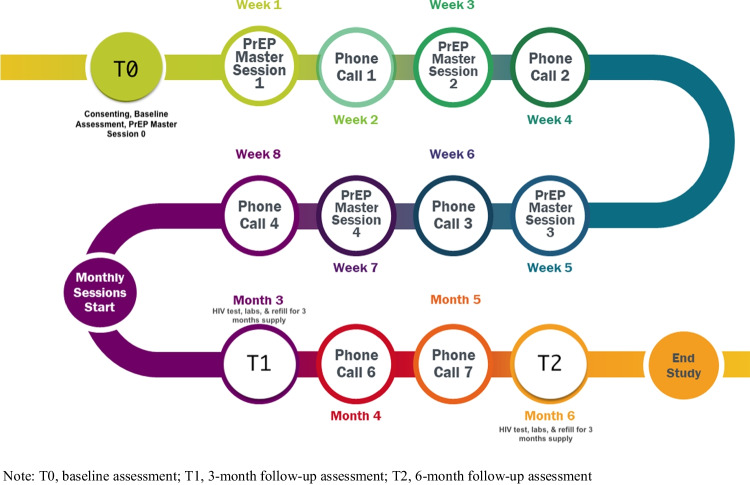
Intervention timeline. Note: T0, baseline assessment; T1, 3-month follow-up assessment; T2, 6-month follow-up assessment

Figure 1 gives an overview of the pilot intervention which utilized a one group pretest, post-test design, with repeated measures over time. Overall, the intervention consisted of 7 research visits over a 6-month period—an introductory session at baseline (T0), 4 biweekly, face-to-face educational sessions led by the PrEP Master, and 3 visits answering research survey questions (baseline (T0), 3 months (T1), and 6 months (T2)). Each face-to-face session, conducted over an 8-week timespan, was built upon the previous session. After completion of session 4, process evaluations were conducted to obtain feedback from the participants’ perspective. Sessions, 30–60 min in length, covered the importance of daily adherence, proper male and female condom use for STI prevention, gauging and reducing alcohol use, heightened importance of PrEP while engaging in risky behaviors (e.g., substance use), and debunking misconceptions about PrEP and its side effects. Between the biweekly sessions, the PrEP Master conducted biweekly 5-to-10-min check-in calls to encourage adherence, assist with difficulties, and assess PrEP experiences.

All intervention session and process evaluation data were recorded by the PrEP Master. Cross-sectional surveys, about 1 h in length, were administered at T0 and T1, and were conducted using a computer-assisted personal interview created in the Questionnaire Development System (QDS). The measures included questions about healthcare utilization, HIV knowledge and stigma, PrEP knowledge, attitudes, and beliefs, childhood trauma, intimate partner violence (IPV), alcohol and drug use, perceived barriers and facilitators of PrEP adherence, condom perceptions, and sexual behaviors. Participants were offered a modest cash incentive to compensate them for their time, which totaled $270 over the 6-month intervention period.

### Measures

#### Demographic Characteristics

Demographic data collected from the sample included age, race/ethnicity, marital status, education, insurance status, housing, income, incarceration history, and psychiatric institutionalization history.

#### Main Outcome—Sexual

##### *Sexual Risk*

Vaginal episode equivalent (VEE) is a risk index used to measure sexual behavior risk [28–31]. The VEE score incorporates counts of unprotected vaginal, anal, and oral sex and assigns a greater weight to anal sex than vaginal sex and allows for some contribution from oral sex. The formula is as follows: (no. of unprotected vaginal acts + 2(no. of unprotected anal acts) + 0.01(no. of unprotected oral acts).

#### Other Outcomes—Behavioral

##### *Alcohol Use*

The Alcohol Use Disorders Identification Test (AUDIT) [32, 33] is a widely used 10-item screening tool used to assess alcohol consumption, drinking behaviors, and alcohol-related problems. AUDIT scores range from 0 to 40 and classify participants as having low risk (0–7), hazardous (8–15), possible harmful (16–19), or possible dependent drinking (20 +).

##### *Drug Use*

The Drug Abuse Screening Test (DAST-10) [34, 35] is a brief 10-item dichotomous (yes/no) screening tool with high internal consistency used to assess drug use, not including alcohol or tobacco use, in the past 12 months. DAST scores range from 0 to 10 and classify participants as no reported drug problem (0), a low-level problem (1–2), a moderate problem (3–5), a substantial problem (6–8), or a severe problem (9–10).

##### *Substance Use and Sex*

Information on drug and alcohol use proximal to sex was collected using 5 items that queried the use of drugs and alcohol before or during sex in the past 12 months. Each question required a yes (1) or no (0) response and scores ranged from 0 to 5. Higher scores represented higher risk-taking behaviors. An example of a question in this measure was “Thinking back to when you had sex in the past 12 months (or the last time you had sex), had you been drinking alcohol before or during sex?”.

#### Process Evaluations

The full process evaluation tool (found in Appendix 1) was developed by the research team and took about 20 min to complete. It consisted of 16 questions which assessed participants’ comfort discussing PrEP, PrEP use motivations, satisfaction, intentions, and barriers, as well as intervention participation motivations and perceptions. Most questions (*n* = 14) were open-ended to encourage participants to expand on their thoughts. One question asked participants to rate the degree of helpfulness of 13 various intervention aspects from 1 (not at all helpful) to 7 (very much helpful), and 1 question asked participants to select which of the 13 aspects of the intervention they found most helpful.

### Data Analysis

#### Quantitative

Cross-sectional survey data were exported from QDS directly into SPSS Version 23, which was used for all analyses. The significance level for all statistical tests was set at *p* < 0.05. Little’s test using estimation maximization was conducted to determine the type of missingness observed in the dataset [36]. Listwise deletion, an unbiased method of handling missing data, was used to treat cases missing completely at random [37]. The Shapiro-Wilks test was performed to determine whether data were normally distributed [38].

Descriptive statistics were reported for demographic characteristics and other variables. Central tendencies of continuous variables were reported as means (with standard deviations) if the data were normally distributed, or as medians (interquartile ranges) if not; non-parametric tests were used as appropriate. Categorical variables were reported as frequencies (percentages). To identify variables that predicted attrition, chi-square, Mann–Whitney *U*, and independent *t*-tests were used to compare demographic characteristics and outcome scores between women who discontinued the intervention (only completed T0) and women who were retained (completed T0 and T1). Related samples Wilcoxon signed-ranked tests and paired *t*-tests were used to assess the changes in behavioral and psychosocial scores between pre- (T0) and post- (T1) intervention time points. To determine whether sexual risk outcomes differed across behavioral risk levels, the median score changes in VEE were observed in 3 behavioral risk categories based on their baseline risk assessments. Profiles included high vs. low alcohol use, drug use, and substance use proximal to sex. As an exploratory pilot study with the goal of exploring estimates of feasibility, acceptability, and preliminary efficacy to inform implementation of a larger scale intervention, sample size determination using power calculation was not appropriate.

#### Qualitative

Select questions from the 16 items in the process evaluation were analyzed to assess participants’ motivations for PrEP use and acceptability of the intervention (Table 1). Descriptive statistics and frequencies were used to analyze the two questions that were not open-ended. Written responses from paper evaluations were transferred verbatim into Qualtrics. A second researcher reviewed the Qualtrics database against the paper evaluations to ensure responses had been entered verbatim. Data were then exported to Excel for analysis. An inductive approach to thematic analysis was utilized to analyze evaluation transcripts. This data-driven form of thematic analysis provides a flexible approach to analyzing qualitative data through an iterative process allowing for codes to arise as investigators familiarize themselves with the data [39].

**Table 1:** Select process evaluation questions

Domains	Select questions
PrEP use barriers	What were some of the difficulties or barriers you had to overcome in order to take PrEP? What, if any, are ongoing difficulties you currently face?
PrEP use facilitators	When you think about your decision to get a prescription and start taking PrEP, is there one thing that stands out in your mind that led to you doing it? In thinking about your starting to take PrEP, what has helped you make taking PrEP become a daily habit?
Overall intervention perceptions	What did you like about participating in the study? What parts did you dislike about participating in the study?
Perceptions of intervention aspects	For the next items, please use the scale below [from 1 (not helpful) to 7 (very helpful)] to rate the degree to which you found the following information helpful. Which of these (1–13) was the most important to you?

*Full process evaluation questionnaire in Appendix* 1

The six steps for conducting thematic analysis of qualitative data as outlined by Braun and Clarke [39] were followed to analyze the evaluation transcripts. The initial four steps, i.e., (1) familiarization with data, (2) generating codes, (3) searching for themes, and (4) reviewing themes, were conducted independently by two reviewers. Final decisions about defining and naming the themes (step 5) were made jointly by the two reviewers who originally coded all the data independently. A third reviewer assisted in the naming phase in the case of themes that could not be agreed upon by the initial reviewers. The final analysis and write up (step 6) can be found in the “Results” section. In the exemplar quotes extracted to support the distilled themes, text was minimally adjusted to improve understanding. Table 2 includes details of the transcription conventions applied.

**Table 2:** Transcription conventions

Conventions	Reason for application
[word]	text added for clarity
w*rd	censoring an impertinent word
…	Text not directly related to the topic omitted from quote

*All exemplar quotes are direct quotes except quotes containing these conventions.*

## Results

The resulting *p*-values from the Shapiro–Wilk test were < 0.05 for behavioral outcomes and > 0.05 for psychosocial outcomes, indicating that all response data were not normally distributed.

### Participant Enrollment

**Fig. 2:**
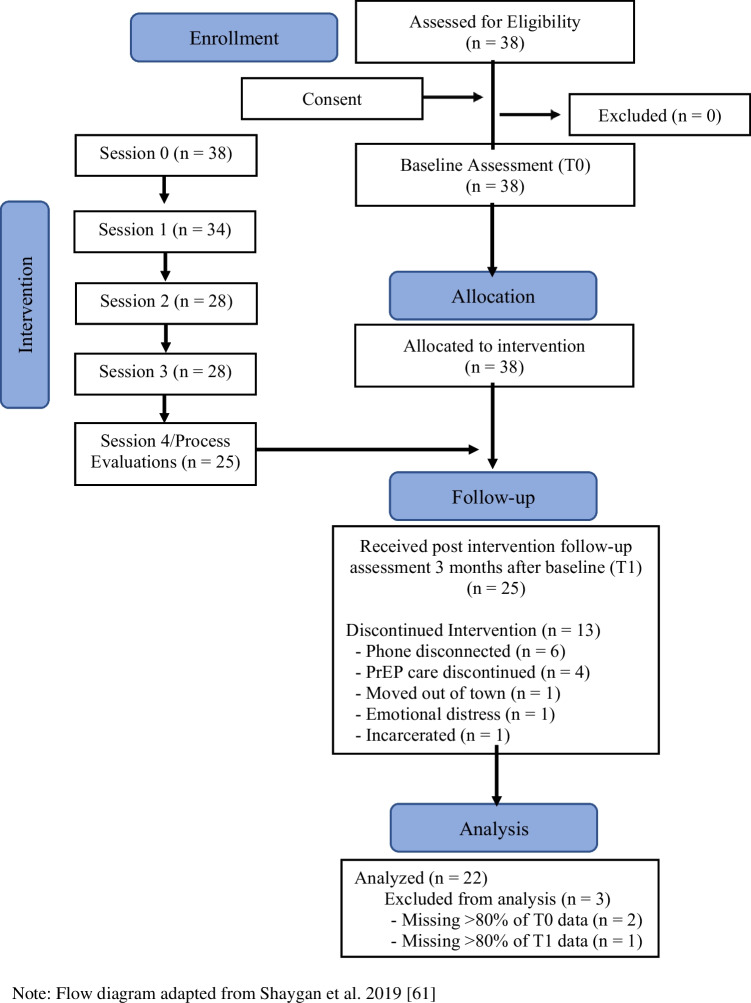
Flow diagram of participant enrollment. Note: Flow diagram adapted from Shaygan et al. 2019 [61]

Figure 2 provides details of the flow of participants through each stage of the study. Overall, 38 women were enrolled between May 2019 to May 2021. Approximately 65% (25) of the women enrolled, received the full intervention—sessions 0 to 4. Of those 25 women, responses from 22 women who completed both the T0 and T1 assessments were included in the final quantitative analyses; process evaluation data for all 25 were included in qualitative analyses. Among the 13 women who discontinued the intervention prior to T1, the majority (77%) did so because their phones had been disconnected or their PrEP care had been discontinued.

### Participant Characteristics

Table 3 provides the baseline demographic characteristics of all study participants (*n* = 38) and the participants who completed T1 (*n* = 22). The mean age of the women was 31 years (*SD* = 7.208) with a range from 19 to 44. The majority of the sample were single, non-Hispanic Black women with African American (76.3%) being the most prevalent ethnicity reported. Women were largely unemployed and low-income (e.g., making less than $10,000 per year) with about 80% having a high school diploma or less. A large proportion reported past homelessness, incarceration, and psychiatric or substance abuse facility institutionalization.

**Table 3:** Characteristics of women enrolled in the intervention

	Baseline (T0)			3-month follow-up (T1)	
	*M*	*SD*	*M*		*SD*
Age (years)	31.21	7.208	32.14		6.875
	Frequency	%	Frequency		%
**Race**					
Black	37	97.4	22		100
White	1	2.6	0		0
Hispanic origin/descent					
Non-Hispanic	34	89.5	20		90.9
Hispanic	4	10.5	2		9.1
Ethnic background					
African American	29	76.3	16		72.7
Haitian/Haitian American	4	10.5	3		13.6
Dominican	2	5.3	1		4.6
Other	3	7.9	2		9.1
Education					
Some high school or below	18	47.4	9		40.9
High school graduate/GED	15	39.5	10		45.5
Some college	4	10.5	2		9.1
College graduate or above	1	2.6	1		4.5
Marital status					
Single/never married	30	78.9	18		81.8
Divorced/widowed/separated	6	15.8	3		13.6
Married	2	5.3	1		4.6
Employment status					
Unemployed	23	60.5	16		72.7
Employed	15	39.5	6		27.3
Health insurance status					
Insured	29	76.3	17		77.3
Uninsured	9	23.7	5		22.7
Income					
≤ $9,999	28	73.7	15		68.2
$10,000 to $19,999	5	13.2	3		13.6
≥ $20,000	5	13.2	4		18.2
Have you ever been…					
Homeless	24	63.2	12		54.5
Incarcerated	21	55.3	13		59.1
In a behavioral health facility	16	42.1	7		31.8
Drug use history					
Non-medical drug use	7	18.4	2		9.1
Marijuana use in the past 30 days	20	52.6	6		27.3

T0 *N* = 38; T1 *N* = 22; *M* mean, *SD* standard deviation

Tables 4 and 5 show results for the comparisons between discontinued and retained participants. Analyses to identify predictors of attrition yielded education level (*U* = 108, *p* = 0.03, η^2^ = 0.13) as the only statistically significant factor. Women who were retained through T1 were significantly more educated than women who discontinued prior to T1. Despite the inability of the tests to show statistical significance in other demographic variables between timepoints, mean rank scores showed that women who discontinued were younger (18.84 vs. 19.98), had higher drug use (19.32 vs. 17.98), had higher alcohol use (19.18 vs. 18.07), and reported lower sexual risk (15.79 vs. 20.23) than women who were retained. The remaining analyses were only conducted among the 22 women retained in the study through T1.

**Table 4:** Baseline demographic characteristics based on retention status

	Discontinued		Retained				
Variables	*n*	%	*n*	%	*χ*^*2*^	*df*	*p*
Employment status					0.212	1	0.65
Unemployed	9	23.7	14	36.8			
Employed	7	6.3	8	21.1			
Health insurance status					0.026^**†**^	1	0.87
Insured	12	31.6	17	44.7			
Uninsured	4	10.5	5	13.2			
Ever been homeless					0.371	1	0.54
Yes	11	28.9	13	34.2			
No	5	13.2	9	23.7			
Ever been incarcerated					0.011	1	0.92
Yes	9	23.7	12	31.6			
No	7	18.4	10	26.3			
Ever been institutionalized					2.268	1	0.13
Yes	9	23.7	7	18.4			
No	7	18.4	15	39.5			
Marijuana use (past 30 days)					0.585	1	0.44
Yes	10	26.3	11	28.9			
No	6	15.8	11	28.9			

* *p* < 0.05; *df* degrees of freedom, *χ*^*2*^ chi-square test statistic

^†^Likelihood ratio; discontinued (*n* = 16); retained (*n* = 22)

**Table 5:** Baseline characteristics and behavioral outcomes based on retention status

	Discontinued		Retained			
Variables/measures	*n*	*M* Rank	*n*	*M* Rank	*U*	*p*
Demographic						
Age	16	18.84	22	19.98	165.0	0.76
Education	16	15.25	22	22.59	108.0	0.03*
Income	16	19.03	22	19.84	168.5	0.81
Behavioral						
VEE	14	15.79	22	20.23	116.0	0.22
AUDIT	14	19.18	22	18.07	144.5	0.76
DAST	14	19.32	22	17.98	142.5	0.68
Substance use proximal to sex	14	19.93	22	17.59	134.0	0.53

* *p* < 0.05; *df* degrees of freedom, *M* mean *U* Mann–Whitney *U* test statistic

### Quantitative—Preliminary Efficacy

#### Risky Behaviors

Baseline DAST scores showed that 18.2% of women retained in the intervention had moderate to severe drug use issues and AUDIT scores showed that 22.7% had some level of problematic alcohol use. Roughly 60% (*n* = 13) of the sample reported using drugs and alcohol proximal to sexual activity. Table 6 shows the results of the test conducted to assess the impact of the intervention on behavioral outcomes (i.e., unprotected sex, alcohol use, drug use, and substance use proximal to sex). Of the four measures included in the analyses, statistical significance was only found in the change of women’s AUDIT scores (*Z* =  − 3.02, *p* = 0.003, η^2^ = 0.41)—indicating a significant decrease in alcohol use scores between T0 and T1.

**Table 6:** Changes in sexual risk, alcohol use, and drug use scores overtime

Measures (range)	Median scores at timepoints		Statistics	
	T0	T1	*Z*	*p*
Sexual risk behaviors				
VEE	9.64	8.55	− 0.426	0.670
Substance use proximal to sex	7.00	6.33	− 1.485	0.138
DAST (0–10)	8.06	6.75	− 0.787	0.431
AUDIT (0–40)	7.38	2.50	− 0.3.020	0.003*

*p < 0.05; *Z* Wilcoxon matched-pairs signed-rank test statistic; *N* = 22

Additional Wilcoxon signed-rank tests conducted to assess whether there were significant changes in sexual risk scores within behavioral risk profiles found no statistically significant changes in median scores (Table 7). However, a notable change was seen in the median sexual risk scores of high-risk women (*n* = 13) who reported using alcohol and drugs before or during sex. In this group, median sexual risk scores decreased from 5.25 at baseline to 2.25 post-intervention (*Z* =  − 1.893, *p* = 0.06). This reduction by over 57% (from 5.25 to 2.25) of median sexual risk scores approached, though did not achieve, statistical significance.

**Table 7:** Changes in sexual risk over time based on behavioral risk profiles

		Median VEE scores at timepoints		Statistics	
Risk profiles	*n* (%)	T0	T1	*Z*	*p*
Alcohol use (AUDIT)					
Low risk	17 (77.3)	6.90	6.21	− 0.353	0.72
High risk	5 (22.7)	3.00	1.00	− 1.461	0.14
Drug use (DAST)					
Low risk	18 (81.8)	8.08	7.94	− 0.653	0.51
High risk	4 (18.2)	0.00	1.00	− 1.000	0.32
Substance use proximal to sex					
Low risk	9 (40.9)	5.25	3.75	− 0.420	0.67
High risk	13 (59.1)	5.25	2.25	− 1.893	0.06

*Z* Wilcoxon matched-pairs signed-rank test statistic; *N* = 22

### Qualitative—Process Evaluation

The mean rating score for helpfulness of the intervention information was 6.58 out of 7. Most women found, “receiving information about PrEP that is geared to a women’s needs and interests,” as the most important aspect of the intervention. “Face-to-face individual sessions to learn about PrEP” and “having another woman to guide me through it” had the second most votes as most important aspects of the intervention. Derived themes provided contextual information not identified in the survey. Overall, 8 themes were identified across three domains (i.e., PrEP use barriers, PrEP use facilitators, and perceived intervention benefits) in the analysis of process evaluation data (Table 8).

**Table 8:** Exemplar quotes supporting themes

Domains	Themes	Quotes
PrEP use barriers	Individual barriers	“Initially scared because of not knowing how it worked but took it because of parents.”
		“Nothing other than oversleeping.”
	Interpersonal barriers	“None, other than my baby daddy trying to make me stop because of side effects.”
		“Nothing really other than my doctor’s hesitancy, thinking it was for gays or I couldn't [take PrEP] because I was pregnant.”
	Structural barriers	“Not having a car and changing phone numbers.”
		“Not getting my refills on time.”
PrEP use facilitators	Individual facilitators	“Knowing that it's an extra layer of protection and my pill planner.”
		“… I took it with my iron daily and sat them next to each other.”
	Interpersonal facilitators	“My fiancé repeatedly reminding me to take it until it became a habit.”
		“When I go outside to look at the impact HIV has had on people in my neighborhood [Liberty City]. I know it's important. I know so many people that died from the virus.”
Perceived intervention benefits	Educational benefits	“Having control over my status. I learned a lot.”
		“Learned a lot that I didn't know about HIV and the medication [PrEP].”
	Therapeutic benefits	“Talking to you and relieving stress. I need someone to talk to.”
		“Talking to someone. It's like therapy and learning about HIV.”
	Financial benefits	“Money & learning about my health.”
		“It was easy and confidential. You're [PrEP Master] friendly and the money was the icing on the cake.”

### PrEP Use Barriers

Barriers were any person, place, or thing that women felt negatively influenced or hindered their utilization of PrEP. A little less than half of the women reported experiencing no difficulties or barriers that had to be overcome to take PrEP. Women who did experience barriers to PrEP use shared barriers that fell into three broad themes—individual, interpersonal, and structural.

#### Individual Barriers

Individual-level barriers mentioned by women were limited PrEP knowledge, self-efficacy issues, and experiencing side effects. One woman shared having to overcome the fear she felt due to lack of knowledge about PrEP stating she was,

*Initially scared because of not knowing how it worked but took it because of parents.*

For context, both of this participant’s parents died of AIDS when she was 17. Knowing someone who died from HIV/AIDS appeared as a salient PrEP initiation motivator and PrEP use facilitator in this sample. Barriers included women’s beliefs about their ability to take PrEP daily due to factors like not remembering, recreational drug use, and oversleeping. The woman who shared that she had to overcome,

*Nothing other than oversleeping.*

essentially her belief that PrEP had to be taken at a certain time of day. Most women reported experiencing nausea as a side effect. One woman reported having to overcome more severe side effects—stating that she experienced “Headaches and fainting spells….” The issue of side effects seemed to be a common factor.

#### Interpersonal Barriers

Most women shared experiences of their PrEP use being hindered by specific people in their social networks. One woman shared that her decision to initiate PrEP was delayed due to.

*The negative things on the [inter]net about side effects.*

Another woman shared how she was negatively influenced by her partner who did not support her decision to take PrEP and who constantly attempted to make her stop taking it. When asked what barriers she had to overcome to take PrEP she said,

*… my baby daddy trying to make me stop because of side effects.*

Multiple women expressed lack of support from their sexual partners in their responses. One woman had to overcome issues with her partner in conjunction with the stigma surrounding PrEP use. The barriers she had to overcome were,

*Hiding it from my baby daddy and being looked at as a whore.*

Possibly in an attempt to avoid being stigmatized, one woman shared an ongoing difficulty to be,

*Not sharing [about PrEP] with my partner.*

Another woman’s response indicated that the limited knowledge her provider had of PrEP was an obstacle that she had to overcome to take PrEP. When she sought out PrEP from her healthcare provider, she was met with resistance. She shared having to overcome,

*… my doctor’s hesitancy, thinking it was for gays or I couldn't [take PrEP] because I was pregnant.*

#### Structural Barriers

Women mentioned a variety of structural barriers that they had to overcome to take PrEP. These included issues with health insurance, getting PrEP prescription refills, transportation, and phones. Structural barriers were not only reported as difficulties that women had to overcome, but also as the most common issue posing ongoing barriers to PrEP use. One woman shared that the difficulties she had to overcome to take PrEP were,

*Not having a car and changing phone numbers.*

Both factors she mentioned could impact her ability to attend or schedule PrEP maintenance appointments and pick up PrEP refills from the pharmacy.

### PrEP Use Facilitators

Women were asked to think about what helped taking PrEP become a daily habit. Responses were related to self-efficacy supported by a variety of reminders and knowledge, attitudes, and beliefs.

#### Individual Facilitators

More than half of the women reported having some sort of reminder that helped them adhere to their PrEP daily. For some, use was facilitated because they already had an established routine since they were taking other medications daily. One woman shared,

*I just wanted to try and test it out to see if it really worked. I took it with my iron daily and sat them next to each other*.

For other women, the use of reminder tools like alarm clocks and pill planners was helpful:

*My alarm helped me know to do it by heart*.

Multiple women described how their knowledge and beliefs about PrEP’s ability to prevent them from getting HIV helped them to take PrEP daily as prescribed. The words “knowing” and “protect” were repeated by several women indicating that they were confident in the idea and truly believed that PrEP could protect them from HIV if they took it daily. One woman noted it was helpful,

*Knowing that I'm safe and secure from catching HIV.*

For many women, a combination of factors facilitated their adoption of daily PrEP adherence. An example of a combination of factors supporting the adoption of daily PrEP use is reflected in the following statement from a woman who shared,

*Knowing that it's an extra layer of protection and my pill planner.*

The idea of individual sexual risk came up multiple times in the domain of cues to action, but not much as a facilitator of PrEP use. Although many women identified individual-level PrEP knowledge- and belief-related factors such as, “Wanting to prevent HIV…,” “Knowing that I’m safe…,” “Thinking about my safety…,” and “The fact that it is protecting me…” as individual factors promoting daily adherence, only one woman mentioned her personal sexual risk:

*Wanting to prevent HIV knowing I have multiple sex partners.*

#### Interpersonal Facilitators

There were not very many women who discussed interpersonal factors facilitating their adoption of daily PrEP use. An interpersonal level facilitator that mirrored responses regarding motivations to initiate PrEP was knowing someone who had died from HIV. One participant stated,

I don't want to die with that sh*t if I can prevent it. I can't help if I get cancer, but I can prevent AIDS. I've lost loved ones to it, and I know how detrimental it can be. My uncle died.

Another interpersonal level facilitator was living in a high HIV-prevalence area. One woman stated,

*When I go outside to look at the impact HIV has had on people in my neighborhood [Liberty City]. I know it's important. I know so many people that died from the virus.*

Another participant discussing what interpersonal level factor helped her adhere to PrEP daily said,

*My fiancé repeatedly reminding me to take it until it became a habit*

For context, during the conversation, this participant shared that her sexual partner was living with HIV with an undetectable viral load. The adherence support that he provided her with by reminding her to take her medication daily helped her develop a habit that he had already adopted. In her case, being with an adherent partner living with HIV was a protective factor—reducing the impact of stigma and providing her with support.

### Intervention Benefits

Analysis of the question “What did you like about participating in the study?” revealed three themes, educational, therapeutic, and financial benefits, under the overarching domain of perceived intervention benefits.

#### Educational

Over half the sample noted that they liked learning as part of the intervention. Multiple women used the phrases “I learned a lot” and “I learned more” when describing their experience. Many women referred to the intervention sessions based in motivational interviewing as “the talks” and described them as fun, interesting, and educational. Most mentioned liking that their participation in the study offered them the opportunity to learn more about HIV and the medication. One woman’s reflection of the experience was,

*I learned a lot that I didn't know about HIV and the medication [PrEP].*

#### Therapeutic

About one-third of the sample referenced talking as part of their highlights of study participation and 20% mentioned specifically enjoying the intervention instructor—the PrEP Master. Although learning was the most frequently reported aspect of the intervention many women found the sessions, or “the talks” as they liked to call them, to be therapeutic, for example,

*Talking to you [PrEP Master] and relieving stress. I need someone to talk to.*

Women used sessions as an opportunity to be listened to. Many revealed childhood traumas for the first time in their lives—things they had never said out loud or things that no one believed when they said them as children. Another participant shared that she enjoyed,

*You [PrEP Master] and the talks. I told you you're my therapist.*

This constant mention of wanting to have and enjoying having someone to talk to uncovered an underlying issue of unaddressed mental health needs in this population, a need for safe places to discuss aspects of life beyond medication adherence, side effects, and benefits. One woman shared that she liked,

*Talking about a lot of things other than PrEP.*

possibly referring to other aspects of the intervention which were all in the context of empowering women to take control over their sexual health.

#### Financial

Multiple women brought up the cash incentive they received for attending study visits when discussing what they liked about participating in the study. Apart from one woman, everyone indicated that they enjoyed getting paid in conjunction with other things. Their responses suggested that the incentive for participation was sufficient, but not coercive. One woman stated,

*It was easy and confidential. You're [PrEP Master] friendly and the money was the icing on the cake.*

Women were also asked about what parts they disliked about participating in the study. Most women shared that there was nothing that they did not like. Instead of listing things they disliked, they said things like “…the study was fine,” “…I learned a lot,” and “I don’t see anything, I was well compensated” alluding to the educational and financial aspects of the intervention that they identified as beneficial. However, 2 out of the 25 women expressed disliking specific intervention aspects. One shared that she did not like, “Having to come into the clinic. Virtual would have been best,” and the other said that she disliked “The amount of time it [an intervention session] takes based on my anxiety and I have kids.” Although their sentiments were not in line with majority of respondents, it is important to include their concerns as factors to consider when incorporating evaluation feedback into the intervention.

## Discussion

This study assessed the preliminary efficacy of the “Talking PrEP with WOC in Miami” intervention that aimed to reduce sexual risk behaviors and encourage PrEP adherence in at-risk minority women who recently initiated PrEP. Although this study did not find significant changes in sexual risk or drug use post intervention, we found that alcohol use significantly decreased in women after intervention participation. A systematic review exploring the association between alcohol use and sexual risk behaviors among Black women found that even non-abusive levels of drinking increased sexual risk-taking in Black women of all ages [3]. This finding suggested that interventions reducing alcohol use have the potential to reduce sexual risk taking in Black women. Additionally, behavioral risk profiles, which grouped women into categories of high or low risk based on pre-determined score cutoff points for measures of alcohol use, drug use, and substance use proximal to sex, also found no statistically significant changes based on the a priori significance level.

One outcome that was not statistically significant but may be of clinical or behavioral importance was the post-intervention decrease in sexual risk observed in women who reported using substances proximal to sex [40]. A dramatic decline of over 50% was observed in the sexual risk scores of women endorsing this risky behavior. The 5-item measure of substance use proximal to sex contained one question about sex and drug use and four questions about sex and alcohol use—essentially measuring a similar construct as the AUDIT scale from which we observed a statistically significant reduction observed in alcohol use.

### Benefits of the Intervention

This study joins a growing body of knowledge that suggests the use of risk reduction interventions that apply elements of MI to support behavior change and medication adherence is effective [24, 25, 41, 42]. The philosophy behind MI is that individuals approach behavior change with different levels of readiness. The goal is achieved through non-judgmental interviewing in which clients do most of the talking [43]. All participants were able to identify benefits of the intervention—therapeutic benefits being one of the more salient themes. Participants mentioned feeling like the intervention sessions were stress relieving and like therapy—stating they needed someone to talk to and, although women identified learning about PrEP as a study benefit, they enjoyed talking about things other than PrEP. A good relationship, characterized by strong rapport between the interventionist and the participant is a central tenet of behavior change interventions based in MI [43].

Participants in this study often mentioned their appreciation for the interventionist’s approach when discussing what they liked about participating in the intervention. They were comfortable sharing private information about their drug and alcohol use, multiple sex partners, the serostatus of their partners, and other sensitive information about themselves and their loved ones. Findings surrounding participants’ perceived benefits of the intervention and their perceptions of the interventionist are indications that the intervention was implemented as intended—person-centered in nature with the interventionist guiding behavior change in a non-judgmental manner [43]. Moreover, these findings suggest that WOC are in need of social support systems that serve as therapeutic outlets for them to express themselves candidly without fear of judgment.

### Study Retention Barriers

Almost one-third of the women enrolled in the intervention stopped participating prior to taking the post-intervention assessment at 3 months. The largest percentage discontinued after session 1. Most women discontinued the intervention due to lack of consistent phone service which hindered communication about upcoming appointments. A study exploring the determinants of loss to follow-up (LTF) in a sample of 7553 patients in HIV treatment found lack of a phone to be a predictor of risk for LTF [44]. Other reasons for being discontinued as a patient included engaging in dangerous or illegal behavior, incarceration, moving far away from the clinic, and emotional distress caused by the sensitive assessment questions asked about IPV and traumatic childhood experiences.

Comparisons conducted between discontinued and retained participants showed that women were largely heterogeneous in terms of sexual risk, risk behaviors, psychosocial outcomes, and most demographic characteristics at baseline. The only significant difference identified was education level—those retained were more educated than those who discontinued participation. Other observed, but not significant, differences among women who discontinued were younger age, higher drug and alcohol use, and lower sexual risk in comparison to women who were retained. These findings are in line with a previous study that found that higher education and older age (≥ 25) were associated with reduced risk for LTF [44]—with less educated women being at higher risk for LTF [45]. Finding ways to successfully engage those likely to discontinue in PrEP interventions may bolster implementation efforts.

### PrEP Use Barriers

Similar to previous studies, common barriers to PrEP use were low perceived self-efficacy [17, 46], experiencing side effects [15, 16, 22, 47], insurance issues [15, 46, 48, 49], trouble getting prescription refills [16, 48], lack of transportation [17, 49, 50], and lack of social support [11, 15, 17, 46]. Existing research suggests that having social support may influence Black women’s healthcare utilization decisions [15, 17, 46]. Most study participants stated that the lack of support from people in their networks, such as doctors [17] and their sexual partners, were barriers. Key findings from a discussion series with Women’s HIV experts identified provider bias to be a barrier to PrEP use [50]. A study exploring the role of social support on African American women’s preventive care usage found that support from family was associated with lower levels of utilization while support from friends was associated with higher utilization [51]. In this study, women did not discuss whether they had family support. However, many noted having very low support from their sexual partners, indicating that they had to hide their PrEP from their sexual partners in fear that they may be stigmatized as promiscuous [46]. Previous research suggests that male partners specifically play an important role in women’s decisions to initiate and adhere to PrEP [52]. Therefore, increasing peer-level support may enhance PrEP implementation efforts among Black women [47].

### PrEP Use Facilitators

Women shared a variety of factors that facilitated their daily PrEP use. Most studies report that women at high risk for HIV infection often have low perceptions of HIV risk—identifying it as a common barrier to PrEP initiation or adherence [15, 53–57]. However, in this study, perception of sexual risk was another common motivator for PrEP use. Multiple women shared being motivated by distrust in their relationships, as well as the risky and/or unknown sexual behaviors of their sexual partners and other men in their sexual networks. A study that explored perceptions of community level HIV/STI risk in women living in urban communities found that because Black and Hispanic women believed their communities had higher concentrations of high-risk partners; they perceived their risk for HIV to be elevated [58]. The study went further to say that this finding may be associated with self-protective behaviors and could be leveraged to help women accurately estimate their risk for HIV [58]. Another factor that emerged as a facilitator of daily adherence was having a relationship with someone who died from complications related to living with HIV. A study conducted in young adults in Kenya found that having a family member with an HIV-related illness or death facilitated PrEP intiation [59]. This finding was true for many women in the study who shared how the death of their parents, uncles, and others in their neighborhoods motivated them to initiate and adhere to PrEP. However, to our knowledge, there is no US-based data supporting the idea that having a relationship with someone who has died from HIV acts as motivation to initiate PrEP. This gap in US literature could be an area of further exploration in future studies. There were a few US-based studies that indicated that having a main partner living with HIV increased the odds of PrEP adoption [60] and getting information from women living with HIV could motivate PrEP adoption as well [15].

Women in this study reported little to no interpersonal level facilitators of PrEP use. The environment and interpersonal context in which they lived (i.e., poverty, high crime, high incarceration rates, high HIV incidence, etc.) shaped their perceptions of HIV risk and motivated many of them to get on PrEP. However, once on PrEP, there was very little mention of support within their environments that facilitated adherence. This finding indicates that promoting and sustaining self-efficacy for daily PrEP adherence in at-risk populations with minimal social support may be essential for effective PrEP implementation. While a review of PrEP adherence in female sex workers found that the need to adhere to multiple medications was a barrier to PrEP use and adherence [10], this study found it to be a facilitator. Reminders, which included alarm clocks, pill planners, and routines for other medications, were reported to be common facilitators of PrEP adherence in Black women [15].

## Limitations

This study is not without limitations. One notable study limitation is the difference in recruitment/data collection time periods across collected responses. Responses collected after February 2020 may reflect lower income levels due to loss of employment that had an impact on many individuals during the pandemic. Due to the pandemic, there were restrictions on social events and strong recommendations for social distancing. These restrictions may have had an impact on the responses given after February 2020 about number of sex partners, alcohol intake, and perception of risk for HIV because social interactions were limited. Another limitation was the number of women who discontinued the intervention, largely due to structural barriers, reducing our sample size. Also, the sample for this study was sourced from a single health center, limiting the generalizability of the study’s findings.

## Conclusion

Given the elevated risk for HIV in alcohol- and other drug-using Black women living and engaging with sexual partners in high prevalence HIV networks, empowering them with the knowledge and behavioral tools needed to reduce their risk for HIV and providing them with support to overcome barriers to HIV prevention is essential for helping them take control of their sexual health. Although preliminary analyses of the “Talking PrEP with Women of Color in Miami” intervention did not find significant changes in sexual risk, the intervention had a positive impact on alcohol use outcomes among these women.

Evaluation of the qualitative and quantitative pilot intervention data revealed persistent individual, interpersonal, and structural level barriers that hindered not only PrEP utilization but study participation as well. This finding suggests an unmet need for support overcoming barriers in Black/African American women. Efforts should be taken to counteract the misinformation and other barriers that arise mainly at the interpersonal level and leverage the interpersonal factors influencing PrEP initiation (i.e., partner’s sexual risk, high prevalence sexual networks, and losing someone to HIV) to help women ascertain their risk for HIV.

Overall, this study highlights how PrEP implementation interventions for at-risk Black women living in high prevalence areas may benefit from including support for women in three main areas: (1) accurately estimating individual and interpersonal HIV risk to facilitate patient-led decision-making to initiate PrEP; (2) providing opportunities to have person-centered prevention conversations that go beyond medication education and adherence messaging, but serve as a therapeutic outlet to discuss daily stressors and social determinants impacting PrEP use—which could increase their perceptions of social support; and (3) incorporating multilevel approaches that respond to the complex social and structural drivers of HIV in high-risk HIV-negative populations. Providing additional layers of support (i.e., transportation and assistance accessing social services) presents a promising strategy for reducing barriers to PrEP use for minority women, retaining them in HIV prevention care, and improving their health outcomes. Doing so will enhance their capacity to translate the increased knowledge and changed attitudes gained from the intervention into protective actions.

## Data Availability

The datasets used and/or analyzed during the current study are available from the corresponding author on reasonable request.

## Appendix 1

Attachment 1: Full Process Evaluation Tool
